# Effect of topically applied *Saccharomyces boulardii* on the healing of acute porcine wounds: a preliminary study

**DOI:** 10.1186/s13104-016-2012-8

**Published:** 2016-04-11

**Authors:** Jessica Partlow, Anthony Blikslager, Charles Matthews, Mac Law, Joshua Daniels, Rose Baker, Raphael Labens

**Affiliations:** Mid-Atlantic Equine Medical Center, Ringoes, NJ USA; College of Veterinary Medicine, North Carolina State University, 1060 William Moore Drive, Raleigh, NC 27607 USA; North Carolina Comprehensive Headache Clinic, Raleigh, NC USA; North Carolina State University, Raleigh, NC USA; The Ohio State University, Columbus, OH USA; Oregon State University, College of Veterinary Medicine Corvallis, Corvallis, OR USA; Royal (Dick) School of Veterinary Studies and Roslin Institute, Roslin, Scotland, UK

## Abstract

**Background:**

Normal wound healing progresses through a series of interdependent physiological events: inflammation, angiogenesis, re-epithelialization, granulation tissue formation and extracellular matrix remodeling. Alterations in this process as well as the bacterial type and load on a wound may alter the wound healing rate. The purpose of this study was to evaluate the effect of topical *Saccharomyces boulardii* on the healing of acute cutaneous wounds, using a prospective, controlled, experimental study, with six purpose bred landrace pigs.

**Results:**

All wounds healed without apparent complications. Comparison of the mean 3D and 2D wound surface area measurements showed no significant difference between treatment groups as wounds decreased similarly in size over the duration of the study. A significant reduction in wound surface area was identified sooner using 3D assessments (by day 9) compared to 2D assessments (by day 12) (P < 0.001). There was no significant effect of treatment group on the number of multiple isolates or the most common isolates obtained relative to control wounds. There was no histologically appreciable difference between the wounds of the different groups.

**Conclusions:**

Topical application of *Saccharomyces boulardii* does not hasten wound healing or change the wounds’ microbiome under the conditions reported in this study.

**Electronic supplementary material:**

The online version of this article (doi:10.1186/s13104-016-2012-8) contains supplementary material, which is available to authorized users.

## Background

Normal wound healing progresses through a series of interdependent physiological events: inflammation, angiogenesis, re-epithelialization, granulation tissue formation and extracellular matrix remodeling [1]. In the context of abnormal or delayed wound healing, decreased severity as well as duration and intensity of inflammation have been associated with faster healing and less scarring [2]. Therefore, alterations to the levels of pro- and anti-inflammatory cytokines and growth factors such as TGF-β play an important role as they can change the rate of extracellular matrix destruction and wound epithelialization [3–5].

Bacterial colonization of wounds can also contribute to inflammation and slow the wound healing process. The presence of local tissue hypoxia and repetitive ischemia–reperfusion injury, along with the alterations in MMP-9 and TIMP levels, create conditions that promote proteolysis, facilitating bacterial colonization and prolongation of the inflammatory phase of healing [6–8]. Thus control of inflammation and bacterial colonization represent important aspects of successful wound management.

*Saccharomyces boulardii* is lyophilized yeast of interest to the authors for its antimicrobial properties and inhibitory effect on pro-inflammatory cytokines in intestinal tissue [9–11]. The effect on pro-inflammatory cytokines is mediated by its action on nuclear factor κB, and mitogen-activated protein kinases ERK1/2 and p38 MAPK [12]. Its antimicrobial action and that of other yeasts is not well studied but has been suggested to be due to the secretion of hydrogen peroxide and organic acids [13], the ability to competitively inhibit pathogenic bacteria [9, 11] and an inherent antimicrobial effect of *Saccharomyces* derived beta-glucan [14]. Given the potential for bacterial colonization to promote inflammation and the importance of inflammation in wound healing, alterations to the type and degree of bacterial wound colonization likely represent a desirable treatment approach. This concept is further supported by results following application of a similar probiotic to cutaneous wounds thereby reducing inflammation and wound closure times [15, 16]. Given the aforementioned effects and positive results utilizing other yeast it is conceivable that *S. boulardii* represents an effective wound treatment agent.

In the present study, we sought to evaluate the effect of topically applied *S. boulardii* on acute wound healing, using a relevant full thickness porcine skin wound model [17, 18] and previously validated 3D-imaging system [19]. We hypothesized that topically applied *S. boulardii* would result in no adverse events, that treated wounds would have a lower number of commensal and contaminant organisms present, that *S. boulardii* would have an effect on the histopathology of treated relative to non-treated healing wounds, and that *S. boulardii* treated wounds would have a shorter time to closure.

## Methods

Six pigs (three intact females, three castrated males) aged 9 weeks and of mean weight 19.6 kg (± 1.67 kg) were inspected to ensure the absence of scaring on the dorsum. All animals were individually housed in comfort footing open grate pens allowing for nose-to-nose contact for a total of 18 days, including 3 days of acclimation prior to commencement of the study. All pigs were fed twice daily at scheduled times with a complete grain feed according to age and weight. Free choice water was available at all times. Animals were videotaped and assessed by the authors for activity levels, response to playful stimuli, social interaction with the neighboring pigs and food intake to allow intervention if abnormal behavior indicative of stress or pain was noted. The study protocol was approved by the North Carolina State University IACUC committee (reference number 12157).

At study commencement each pig had three full thickness skin wounds created on their dorsum. The procedures were performed under general anesthesia. Flunixin meglumine (1.1 mg/kg IM) and buprenorphine (0.01 mg/kg IM) were administered pre-operatively and anesthesia was induced with a combination of xylazine (0.5 mg/kg IM) and ketamine (11 mg/kg IM). Following orotracheal intubation, anesthesia was maintained with 1–3.5 % isoflurane vaporized in 100 % oxygen. The pigs were positioned in ventral recumbency, and the dorsum of each pig was aseptically prepared. Three circular wounds measuring 4 cm in diameter, with a space of 4 cm between the wounds, were created on the dorsum of each pig with a #10 bard parker scalpel blade using a study template. Hemostasis was achieved with digital pressure and gauze sponges.

**Fig. 1:**
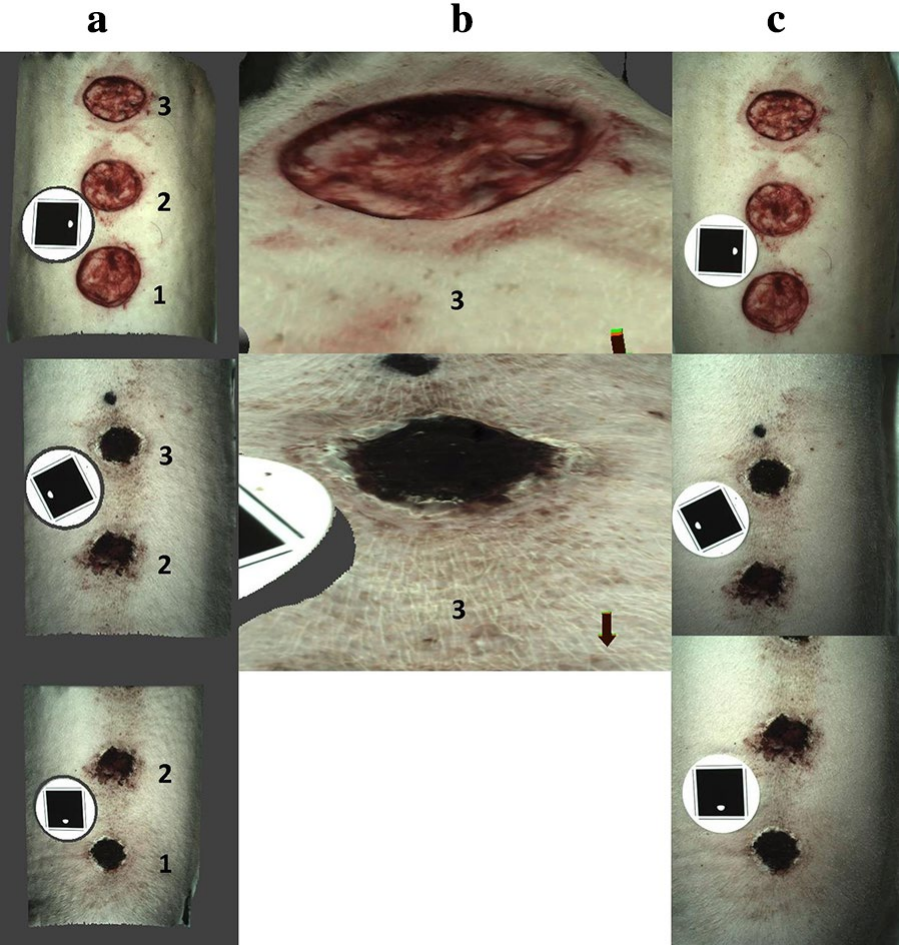
Wounds created on the dorsum of *Pig 2* (wound 1 treated every 3rd day; wound 2 was left untreated and wound 3 was treated once). The wounds’ 3D reconstruction is shown from above (**a**) and for wound 3 also in close-up and side view (**b**) on day 0 (*top row*) and at study completion (*lower rows*). One of three corresponding images used to compute each model is shown in **c** demonstrating the wounds’ truthful simulation. The circular imaging targets necessary for automated model computation and scaling can also be appreciated

In each pig, two of the three wounds were treated topically with a single dose of topical *S. boulardii* (5 billion live organisms, Pure Encapsulations^**®**^, Sudbury MA, USA) at the time of wounding. One of the wounds was then re-treated every 3rd day for a total of five treatments. The remaining third wound served as a control, and received no treatment. Treatment allocation was randomized using a random number generator. Following treatment, wounds were left uncovered and exposed to the environment. The pigs were sedated with xylazine (3 mg/kg IM) to facilitate treatment, visual inspection, and wound imaging every 3rd day, with the study end point being the conclusion of imaging and inspection on day 15, 3 days after the 5th and final wound retreatment. Wounds were imaged using a commercial 3D-wound imaging system, consisting of a dedicated camera, associated software system and single use optical targets allowing automated 3D rendering of wound models and further processing (Eykona Medical US Inc., Greenville, NC, USA) (Fig. 1). Subsequently, similar to a previous report [20] wound margins were traced in generated computer models by one author (RB), to determine the wounds’ surface area (mm^2^) by projection of traces onto a plane surface (2D measurement) or by three dimensional representation thereby taking the wounds’ precise topography into account (3D measurement).

In addition to wound imaging and visual inspection, sterile swabs were used to obtain samples for aerobic culture. This was performed at the time of wounding, study midpoint and day 15. Total number of colony forming units, and the five most prolific bacterial and fungal species were identified for each wound at each time point. Tissue samples (5 mm diameter full thickness biopsies from the center and periphery of the wound) were also obtained for histologic evaluation at the termination of the study on day 15. The tissue samples were fixed in 10 % neutral buffered formalin, routinely processed using an automated tissue processor, embedded in paraffin, sectioned at 5 μm, and stained with hematoxylin and eosin (H&E). The tissue sections were examined and graded by a board certified veterinary pathologist (ML) according to a 5-tiered grading scale, as follows: Grade 1, minimal changes from normal including edema, infiltration of inflammatory cells and overlying serum crust formation, thickening of the epidermis, and accumulation of granulation tissue; Grade 2, mild changes as described above; Grade 3, moderate changes; Grade 4, moderately severe changes, including some areas of necrosis of the epidermis; and Grade 5, severe changes including diffuse edema, marked inflammation, dense granulation tissue in the dermis, and often marked thickening of the epidermis with areas of epidermal necrosis. The board certified veterinary pathologist (ML) was blinded to the treatment groups.

### Statistical analysis

Imaging data was analyzed using Kruskal–Wallis one-way analyses of variance on ranks allowing individual comparisons between measurements (3D vs. 2D), treatment groups and days. When significance was detected the Dunn’s method was applied to perform multiple comparisons versus a control group (day 0 observation). Two-way ANOVA was used to evaluate for any difference between the treatment and control groups in number of CFU after normal transformation of the data. To assess for a difference between the treatment and control groups in numbers of bacterial isolates (binned as either ≤1 or >1 Isolate) Fisher’s Exact Tests were performed. For all statistical tests, completed using SigmaStat^**®**^ software (Systat Software, Inc., San Jose, CA, USA) significance was set at P ≤ 0.05.

## Results

For the duration of the study, all animals remained comfortable and feed consumption remained constant, there was no alteration in activity level or social interaction. All wounds had healed with normal progression and contracted down to a minimal surface area by day 15. There was no appreciable difference observed in wound appearance between the treatment and control groups over the course of the study. Figure 1 shows examples of 3D wound reconstructions (a, b) alongside one of the corresponding digital photographs used in rendering 3D models (c).

**Fig. 2:**
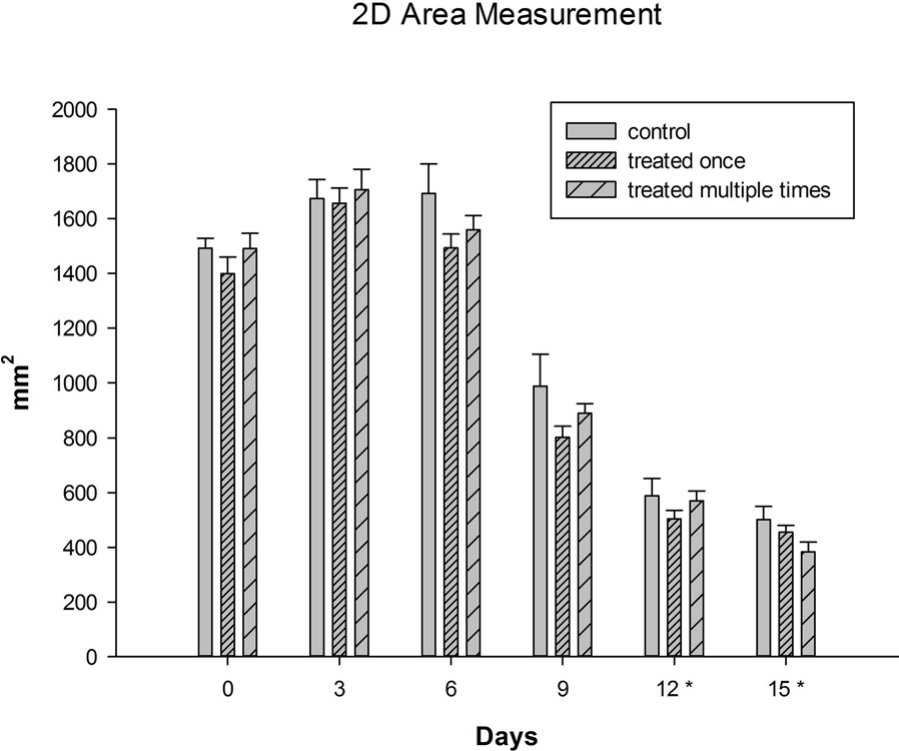
*Graph* of mean wound surface area for control, single and multiply treated wounds at each measured time point; *error bars* represent standard deviations, *asterisks* indicate significant differences from time 0 (P ≤ 0.001)

**Fig. 3:**
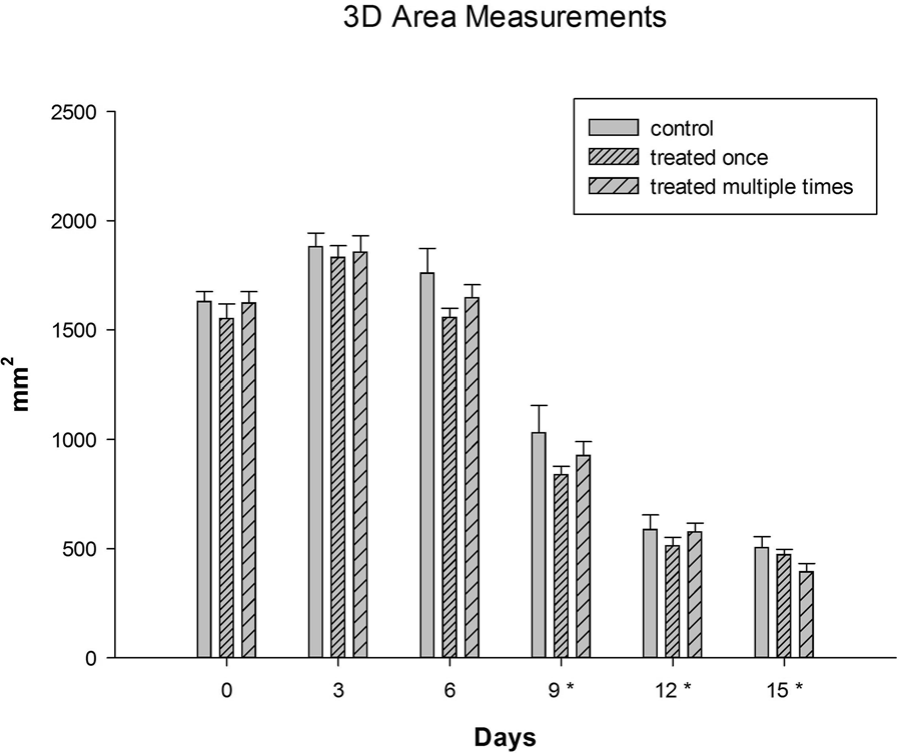
*Graph* of mean wound 3D-surface area for control, single and multiple treated wounds at each measured time point, *error bars* represent standard deviations, *asterisks* indicate significant differences from time 0 (P ≤ 0.001)

Comparison of the mean 3D and 2D wound surface area measurements showed no significant difference between treatment groups as wounds decreased similarly in size over the duration of the study. Regarding the utility of 3D measurements, a significant reduction in wound surface area was identified sooner (by day 9) compared to 2D assessments (by day 12) (P < 0.001) (Figs. 2, 3; Additional file 1).

Organisms other than *S. boulardii* were isolated from all 18 wounds at all time points. There were a total of 41 isolates from the control wounds during the study, and slightly lower numbers from the single (36 isolates) and multiply (37 isolates) treated groups. *Staphylococcus hyicus, Enterococcus* spp., *Bacillus* spp., *Micrococcus* spp., *S. aureus* and *Candida* spp. were isolated from the wounds in this study. *Staphylococcus hyicus* was most commonly isolated (37 isolates) followed by *Staphylococcus aureus* (21 isolates), *Entercoccus* spp. (14 isolates) and *Bacillus* spp. (12 isolates). There was no significant difference in the number of multiple isolates (binned as either ≤1 or >1 isolate) or the most common isolates obtained between the three groups (Additional file 2).

**Fig. 4:**
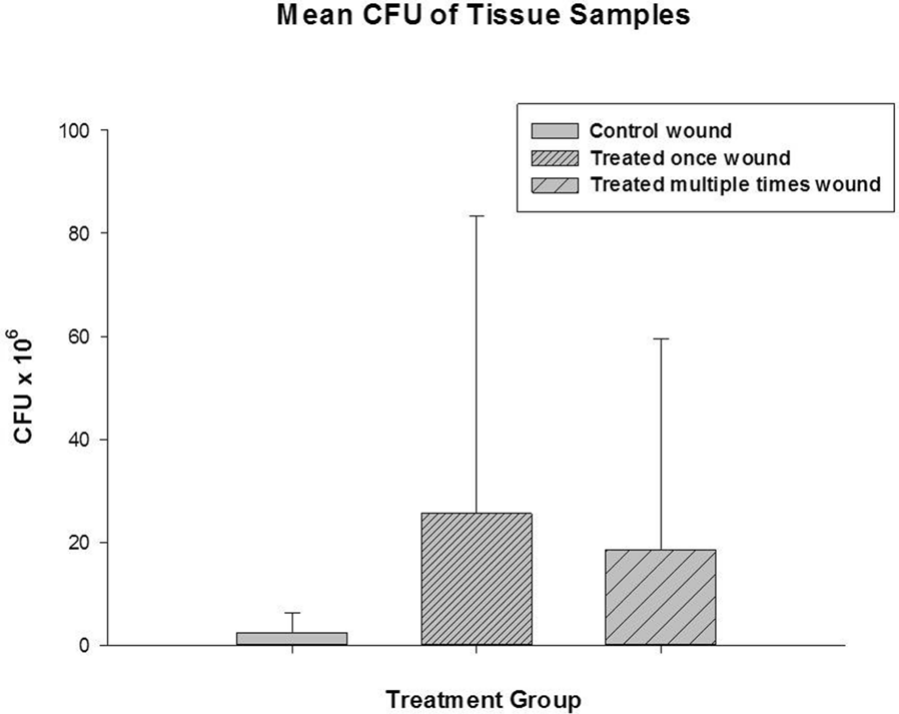
*Graph* of mean CFU eluted from control, single and multiply treated wounds at the final time point (day 15 of study)

Immediately after wound inoculation on day 0 there was a significantly greater number of colony forming units (CFU) from both treatment groups when compared to controls (P < 0.001) (Fig. 4). There was no significant difference in CFU between the three groups at mid or at the final time point of the study.

**Fig. 5:**
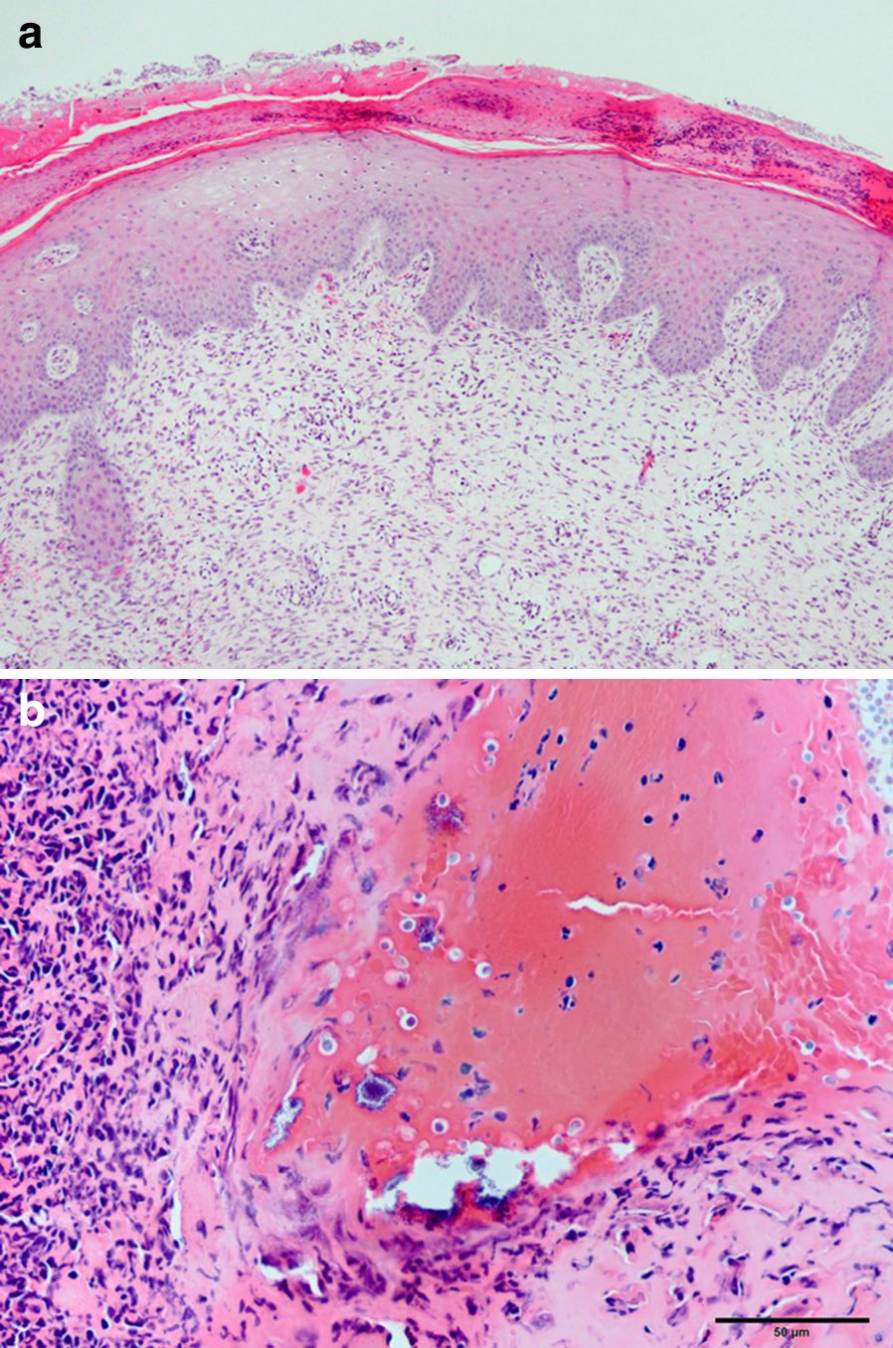
**a** The 5 mm diameter tissue samples from the wounds’ center stained with hematoxylin and eosin were characterized microscopically as having a focal ulcer (ulcer not shown in this photo) with an overlying neutrophilic crust containing *small colonies* of cocci bacteria and numerous yeasts. In **a**, the epidermis is moderately hyperplastic and the dermis is thickened by abundant granulation tissue (associated with wound healing) composed of numerous small blood vessels and thick collagen bundles interspersed with small to moderate numbers of macrophages. **b** Higher magnification photomicrograph showing the superficial epidermis at *left* and *bottom*, which is heavily infiltrated by neutrophils, most of which are degenerate or necrotic. At *top right* is a serocellular crust that contains degenerate neutrophils, erythrocytes, necrotic cell debris, colonies of bacterial cocci, and moderate numbers of yeasts, presumed to be *Saccharomyces boulardii*

Table 1 shows the histological grade for each wound tissue sample in the study. The wound edges were each characterized microscopically as having a focal ulcer with an overlying neutrophilic crust containing small colonies of cocci bacteria and groups of small to numerous yeasts (presumed to be the *S*. *boulardii* yeasts that were applied). The epidermis was moderately hyperplastic, and the dermis all the way to the panniculis was thickened by granulation tissue composed of numerous small blood vessels and thick collagen bundles interspersed with small to moderate numbers of macrophages and scattered perivascular aggregates of lymphocytes and plasma cells (Fig. 5a, b). There were no histologically appreciable differences between the different animals or when comparing the wounds of the different groups.

**Table 1:** Histopathology grading of each studied wound

Pig	Wound	Treatment group	Histologic grade	Comments
1	1	Control	3	
1	2	Multiple treatments	3	
1	3	Single treatment	3	Contains a focal, intense crust with numerous yeasts
2	1	Multiple treatments	3	Large areas of ulceration in these sections
2	2	Control	2	Milder inflammatory reaction in this wound
2	3	Single treatment	2	
3	1	Control	3	
3	2	Single treatment	3	
3	3	Multiple treatments	3	
4	1	Control	3	
4	2	Single treatment	4	
4	3	Multiple treatments	3	
5	1	Single treatment	3	
5	2	Multiple treatments	3	
5	3	Control	3	
6	1	Multiple treatments	3	
6	2	Control	1	
6	3	Single treatment	3

## Discussion

The goal of this study was to evaluate for any treatment effect of topically applied *S*. *boulardii* to acute porcine skin wounds a model allowing wound healing in 12–14 days, with an epithelialization rate of approximately 0.05 mm/h [13].

As a probiotic yeast, *S*. *boulardii* was of interest for its antimicrobial and anti-inflammatory properties [9–11]. Previous studies of probiotics applied to cutaneous wounds have shown decreased inflammation and reduced time to closure [15, 16, 21]. A topically applied nitric oxide producing probiotic has also been shown to improve wound healing outcomes [22]. In contrast to these studies, we found no clear difference in wound healing outcomes when *S*. *boulardii* was applied in either single or multiple applications at doses of 5 billion organisms. Thus we found no evidence to support the clinical application of *S*. *boulardii* to accelerate wound healing but these results should be considered cautiously in light of the small study population and resulting low statistical power. Furthermore doses of yeast included in two similar wound healing studies have been widely variable and it is possible the dose of *S*. *boulardii* may have been inadequate to exert a therapeutic effect [16] [21]. The dose of 5 billion *S. boulardii* organisms was chosen to reflect the dose used safely in previous studies utilizing this organism [23–25].

As measurements obtained on the basis of three dimensional wound imaging are associated with greater precision than conventional techniques [20] a commercial 3D imaging and processing system was used in this investigation. Compared with a self-developed system [20], the authors felt that this previously validated system [19] was easy to operate and produced life-like 3D reconstructions allowing reliable clinical assessments to be made [19]. In addition to the technique’s superior reliability, three dimensional wound imaging also enables quantification of a wound’s topography. Disturbed wound healing may result in irregular and uneven wound beds; 3D imaging is therefore expected to be of great clinical value. Given that in this investigation both 2D and 3D measurements were equally affected by the precision of the generated 3D model the observed difference between the 2D and 3D assessed rate of healing is likely indicative of a change in wound topography prior to wound contraction occurring (Figs. 2, 3). Therefore 3D surface area measurements are likely not only more precise but also allow detection of a treatment effect sooner when compared to 2D surface area assessments.

The process of cutaneous wound healing involves formation of a fibrin and fibronectin matrix followed by inflammatory cell infiltration and wound remodeling. The wounds of both experimental and control groups in this study healed routinely with a crust of neutrophils containing small colonies of cocci bacteria and a granulating wound edge containing macrophages, lymphocytes and plasma cells. This preliminary study of the effect of *Saccharomyces* was performed in an acute model of wound healing. In a more pro-inflammatory state of a chronic non-healing wound there is an altered cytokine profile, with elevated levels of matrix metalloproteinases (MMPs) and reduced levels of MMP inhibitors along with abundant pro-inflammatory cytokines [6]. In such circumstances a treatment effect of *S*. *boulardii* might be observed and future in vitro or in vivo studies on models of chronic wound healing may be considered. In addition given the relevance of pro-inflammatory cytokines future work should include their quantification.

Treatment and control group wounds in our study cultured organisms other than *S*. *boulardii* at similar levels (CFU), and the wounds treated with both single and multiple doses of *S*. *boulardii* grew a similar population of organisms to the control wounds. The exception to this being immediately after initial inoculation of the treated wounds with *S*. *boulardii*, which was reflected in the culture results. These findings indicate a lack of effect of *S*. *boulardii* on the wound microbiome of acute open wounds. These findings could also be due to the *S. boulardii* being no longer live and viable despite it being stored appropriately. It is also notable that this analysis had the highest power of the study (0.96). Future studies may evaluate for an effect of *S*. *boulardii* on models of grossly contaminated wounds. In such wounds, an elevated bacterial load and presence of potentially pathogenic bacterial species may be positively affected by the previously demonstrated inhibitory effect of *S*. *boulardii* [10].

Wound healing investigations have often depended on the use of animal models, including porcine models, since they provide a means of studying the complex interactions occurring in living tissue without some of the limitations and artifacts inherent to in vitro techniques [26, 27]. Wounds were purposefully left uncovered allowing bacterial colonization to occur to evaluate treatments in the context of competitive co-colonization. While it is more typical to cover wounds with a dressing we left the wounds uncovered and exposed to air so as not to alter the microbiome of the healing wounds [28]. The study size the number of wounds evaluated was appropriate for a preliminary study, utilizing adequate controls and including the randomization of treatments. This meant that controls were in varying positions and distanced at least 4 cm from the most frequently treated wounds thereby rendering contamination of control wounds an unlikely but not impossible event. Equal numbers of male and female animals were chosen in an effort to prevent potential bias. Keeping the studies limitations in mind we found that single or multiple applications of *S*. *boulardii* did not have a treatment effect on wound measurements, microbiome or histology but larger numbers may be required to identify such an effect. Additionally, while quantitative wound assessments did not detect a treatment effect, qualitative variables such as wound exudate, bleeding and color, all typically assessed by clinicians were not evaluated in this study. While there are some benefits to including qualitative measures, including a potentially increased ability to identify a treatment effect, the value and degree of scientific evidence provided by these evaluations is often lower and it can be difficult to run statistical analyses on such measures.

## Conclusion

The results of this study indicate a lack of effect of *S*. *boulardii* applied in an acute wound-healing model. Our findings do not support the use of *S*. *boulardii* in a clinical acute wound healing environment. Future investigations may evaluate efficacy in wound healing models that display an altered inflammatory profile and bacterial microbiome and should also assess the effect of *S*. *boulardii* on biomolecules relevant to the wound-healing environment.

## Additional files

10.1186/s13104-016-2012-8 Wound measurements.
10.1186/s13104-016-2012-810.1186/s13104-016-2012-8 Wound microbiology.

## Abbreviations

TGF- β: transforming growth factor β
MMP-9: metalloproteinases 9
TIMP: tissue inhibitor of metalloproteinase
ERK1/2: extracellular-signal-regulated kinases
P38 MAPK: P38 mitogen-activated protein kinases
*S. boulardii*: *Saccharomyces boulardii*
CFU: colony forming unit
